# A missing link in the bench-to-bedside paradigm: engaging regulatory stakeholders in clinical genomics research

**DOI:** 10.1186/s13073-016-0351-7

**Published:** 2016-09-21

**Authors:** Julianne M. O’Daniel, Jonathan S. Berg

**Affiliations:** Department of Genetics, CB # 7264, University of North Carolina, Chapel Hill, NC 27599 USA

## Abstract

For genomic medicine research to be fully translated into clinical care, it is critical for researchers to engage stakeholders who ultimately regulate the use of genomic technologies and therapeutics within healthcare practice. Herein, we describe an example of how this might work.

## Need for stakeholder engagement

Notwithstanding the historical hype of genomic medicine [1], there is growing evidence demonstrating successful clinical applications for genomic technologies, particularly for inherited disease diagnosis [2]. Genomic testing is also increasingly used in clinical cancer management and pharmacogenomics [3, 4].

Producing the evidence is but the first step toward implementation of genomic medicine. Efforts to engage with target communities and patient stakeholders are now a frequent component of clinical genomics research protocols [5]. Successful partnerships have been demonstrated to increase satisfaction, trust, and ultimate uptake of results. However, for the benefits of genomic medicine research to be realized “at the bedside” by the public who funds it, it is critical to address the role of entities that regulate different aspects of healthcare. These roles include both the regulation of the tests and technologies as well as reimbursement or coverage decisions for the utilization of these technologies in clinical practice. These regulatory roles are crucially important to ensure that novel research findings are safe and effective for standard clinical practice. Although there is recognition of the need to engage regulatory stakeholders for clinical genomic research [6], more progress is needed. Perhaps there are lessons to be learned from the structure of successful patient–researcher partnerships which emphasize co-learning and capacity building, iterative processes, dissemination of results among all partners, and commitment to long-term sustainable relationships [7].

Currently, many of the research programs producing the growing body of evidence for healthcare applications of genomic medicine are associated with or directly linked through large-scale consortia funded by the National Human Genome Research Institute (NHGRI), under the purview of the National Institutes of Health (NIH). Examples include Clinical Sequencing Exploratory Research (CSER; https://cser-consortium.org/), Implementing Genomics in Practice (IGNITE; https://ignite-genomics.org/), and Clinical Genome Resource (ClinGen; https://www.clinicalgenome.org/). A primary aim of these public-funded projects is to elucidate whether and how genomic technologies can be utilized to improve patient outcomes and optimize healthcare for the population and individual. In other words, much of research is focused on the analytic and clinical validity as well as clinical utility of genomic technologies. Researchers’ definitions of these concepts may vary considerably from that of regulatory groups, however. Rather than producing scientific evidence with the assumption that it will meet the evidence requirements for policy development, it is important to engage individuals making both coverage and regulatory decisions to understand the evidence needs and assessment processes of these stakeholders. Such engagement may facilitate understanding between researchers and regulators and help ensure study designs and outcomes achieve their intended goals.

## Developing a framework for engagement

The ClinGen program has begun to develop such engagement partnerships. The central aim of ClinGen is to build an authoritative resource that defines the clinical relevance of genes and variants for use in clinical research and medicine [8]. Regulatory groups tasked with assessing the clinical validity of genomic medicine applications have a vested interest in the outcomes of ClinGen. To begin developing the relationship between researchers and external stakeholders, it is important to define who the regulatory stakeholders may include. For ClinGen, these include groups tasked with ensuring the analytic and clinical validity of genomic tests such as those that regulate (1) the development of tests, (2) the assignment of procedural codes used to define intended use of a genomic test, and (3) the decisions regarding the appropriate clinical use of genomic testing via health policy for medical coverage and reimbursement. Additional stakeholders include professional societies who may produce guidelines utilized by regulatory bodies and patients who share access to clinical genome data.

In order to build mutually beneficial relationships, it is then important to explore the various groups’ role(s) in genomic medicine and their evidence/data needs for policy development and decision-making. Key questions may include the following: (1) What is each regulatory group’s role(s) in genomic medicine and how can research efforts contribute to their specific aims? (2) What types of evidence are needed to support each group’s regulatory decisions? (3) Do the research aims align with the evidentiary needs and/or can the study protocol be amended in response to the required data/evidence? (4) Are there mutual benefits to establishing a relationship? (5) Is it feasible to build and sustain a relationship of transparency, communication, and dissemination?

**Fig. 1:**
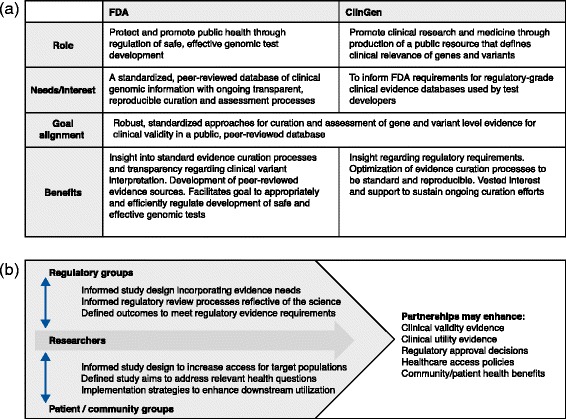
**a** Overview of the regulatory stakeholder relationship established between the Clinical Genome Resource (*ClinGen*) and the United States Food and Drug Administration (*FDA*). The two groups defined their common goals and developed transparent communications to seek insight and share progress. **b** Summary of the dynamics and benefits of partnering with community and regulatory stakeholders

The US Food and Drug Administration (FDA) proactively expressed interest in ClinGen’s approach to curation of genes and variants. Based on this interest, ClinGen sought to establish a process of engagement with the FDA as the first stakeholder. The interactions to date between ClinGen researchers and the FDA described here are presented as an example of how such engagement could work. To facilitate the development of this stakeholder relationship, a small workgroup of key program contacts/liaisons from the relevant organizations (ClinGen, NHGRI, and FDA) met monthly via conference calls. At the outset, roles and interests were defined as summarized in Fig. 1a.

The open dialogue generated by establishing this relationship has provided a means through which to clarify and/or contextualize confusing or misunderstood jargon and concepts in terms of the research and the regulatory processes. Acknowledging the need for shared learning is essential to building a mutually respectful relationship. The formal development of a dedicated liaison and workgroup tasked with engaging regulatory groups has allowed broader contextualization of overarching goals and minimized the possibility for ambiguous messages or distorted perceptions.

The relationship has allowed ClinGen researchers to understand the potential uses of ClinGen resources for FDA regulatory purposes and to align curation processes to produce a resource consistent with FDA draft guidelines on regulatory-grade databases [9]. The accompanying announcement reflected the FDA’s perspective on such relationships as helping to “conceptualize this flexible approach that strikes the important balance between safeguarding public health and promoting innovation” [10].

## Future directions

Through this experience, it has become evident that engaging with regulatory stakeholders, such as the FDA, can not only be helpful in the development of the ClinGen resource, but is essential to guiding appropriate utilization of the generated evidence to support improved health through genomic medicine (Fig. 1b). This experience represents merely the first step in engaging relevant regulatory stakeholders and was simplified as the interaction was focused on a single stakeholder. Ongoing and future efforts to establish interactions with additional stakeholders such as those representing payer and reimbursement perspectives, as well as clinical authorities who may wish to use ClinGen products in making recommendations, present exciting new challenges, as designing studies and outcomes that are responsive to a broader range of decision-makers will require considerable flexibility.

The need for continued and increased regulatory engagement activities among genomic medicine research is great, and the potential impact of these types of interactions to further clinical genomic medicine may be well worth the effort.

## Abbreviations

ClinGen: Clinical Genome Resource
FDA: United States Food and Drug Administration
NHGRI: National Human Genome Research Institute

## References

[1] Caulfield T, Condit C (2012). Science and the sources of hype. Public Health Genomics.

[2] Retterer K, Juusola J, Cho MT, Vitazka P, Millan F, Gibellini F (2016). Clinical application of whole-exome sequencing across clinical indications. Genet Med.

[3] Meric-Bernstam F, Amber J, Holla V, Bailey AM, Brusco L, Chen K, et al. A decision support framework for genomically informed investigational cancer therapy. J Natl Cancer Inst. 2015;107. doi:10.1093/jnci/djv098. 10.1093/jnci/djv098 PMC4651038 25863335

[4] Gammal RS, Crews KR, Haidar CE, Hoffman JM, Baker DK, Barker PJ, et al. Pharmacogenetics for safe codeine use in sickle cell disease. Pediatrics. 2016;138. doi:10.1542/peds. 10.1542/peds.2015-3479 PMC4925073 27335380

[5] Deverka PA, Schully SD, Ishibe N, Carlson JJ, Freedman A, Goddard KA (2012). Stakeholder assessment of the evidence for cancer genomic tests: insights from three case studies. Genet Med.

[6] Horowitz CR, Abul-Husn NS, Ellis S, Ramos MA, Negron R, Supron M (2016). Determining the effects and challenges of incorporating genetic testing into primary care management of hypertensive patients with African ancestry. Contemp Clin Trials.

[7] Israel BA, Parker EA, Rowe Z, Salvatore A, Minkler M, Lopez J (2005). Community-based participatory research: lessons learned from the Centers for Children’s Environmental Health and Disease Prevention research. Environ Health Perspect.

[8] Rehm H, Berg J, Brooks L, Bustamante C, Evans J, Landrum M (2015). ClinGen – the Clinical Genome Resource. N Engl J Med.

[9] Food and Drug Administration. Use of public human genetic variant databases to support clinical validity for next generation sequencing (NGS)-based in vitro diagnostics. Federal Register; 2016. Document no. 16008.

[10] Food and Drug Administration. FDA advances Precision Medicine Initiative by issuing draft guidances on next generation sequencing-based tests. 2016. http://www.fda.gov/NewsEvents/Newsroom/PressAnnouncements/ucm509814.htm. Accessed 5 Sept 2016.

